# Trabecular Variant Juvenile Ossifying Fibroma of the Maxilla

**DOI:** 10.7759/cureus.1684

**Published:** 2017-09-14

**Authors:** Ashwan Paranthaman, Vandana Shenoy, Senthil Kumar, Laavanya Marimuthu, Sakthivel Velusubbiah, Shonali Vijayaraj

**Affiliations:** 1 Oral and Maxillofacial Surgery, Thai moogambigai dental college & hospital; 2 Oral and Maxillofacial Surgery, Boomi's Total Dental Care

**Keywords:** juvenile ossifying fibroma, resection, fibrooseous lesions, maxilla

## Abstract

Juvenile ossifying fibroma (JOF) is a benign, bone-forming neoplasm occurring primarily in children and adolescents. JOF is an aggressive variant of ossifying fibroma of the jaw with a variable clinical behavior and a high tendency for recurrence. Early detection and prompt treatment are required to treat JOF successfully. This case report describes JOF in a 13-year-old girl presenting with a year-long, gradually progressive swelling on the right side of her face with typical clinical, radiological, and histopathological features.

## Introduction

Ossifying fibroma is an uncommon, benign, bone-forming tumor distinguished from fibro-osseous lesions by the age of onset, clinical presentation, and aggressiveness [1]. Ossifying fibroma is divided into conventional and juvenile subtypes. Juvenile ossifying fibroma (JOF) was first described by Sir Benjamin, et al. in 1938 as an “osteoid fibroma with atypical calcification,” and in 1952, Sir Johnson, et al. coined the term “juvenile active ossifying fibroma” [2]. JOF is also known as juvenile active ossifying fibroma, juvenile aggressive ossifying fibroma, trabecular osteodesmoblastoma, and active fibrous dysplasia. JOF is classified as an odontogenic tumor in the second edition of the World Health Organization (WHO) classification of odontogenic tumors [3]. Two percent of all oral tumors in children are JOF, which has an equal predilection for males and females. JOF is found in facial bone (85% of cases), the calvarium (12% of cases), the mandibular region (10% of cases), and, very rarely, an extracranial location (3%) [4]. JOF was further subdivided in a report by El-Mofty into two histopathological variants: trabecular (TrJOF) and psammomatoid (PsJOF). One clinical feature that helps differentiate between the histopathological variants is the site of involvement; PsJOF presents in the paranasal sinuses, and TrJOF presents in the maxilla [1]. 

## Case presentation

A 13-year-old girl reported to the Department of Oral and Maxillofacial Surgery, Thai Moogambigai Dental College and Hospital, Chennai, India, with swelling over the right side of the face for the past year. She reported the swelling was gradually increasing in size over the past four months. The patient has a medical history of an epileptic attack, and she was diagnosed with tubular sclerosis of the brain in 2008, treated with medication since the diagnosis. Her last episode of epilepsy was in 2010 (seven years ago). Family history revealed her mother is also taking medication for epilepsy. The patient also reported a history of blurred vision in her right eye for the past year, and she was prescribed glasses for the high refractive index (amblyopia) in the right eye for correction.

**Figure 1:**
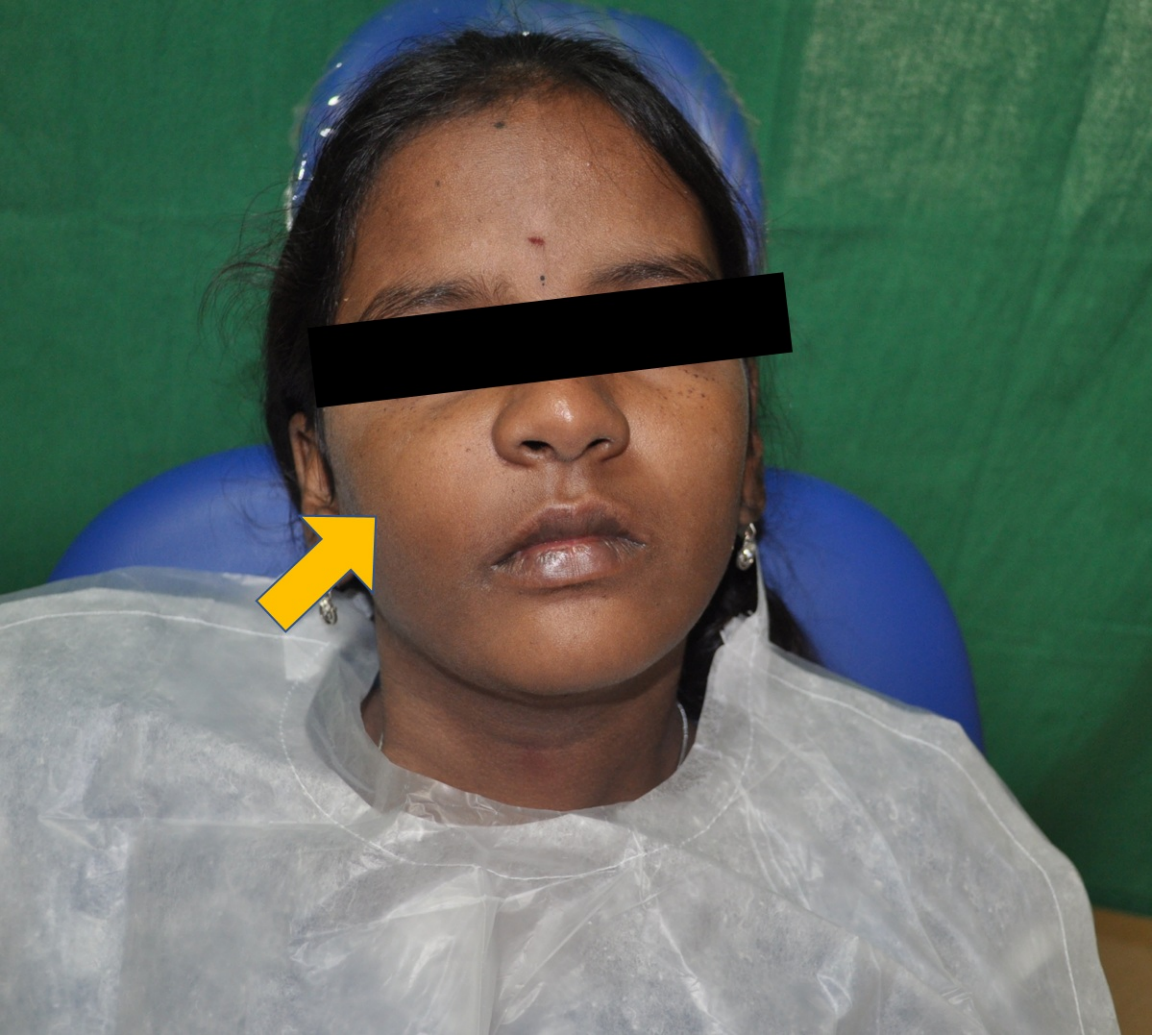
Frontal view of extraoral swelling in the right maxillary region (denoted by the yellow arrow).

**Figure 2:**
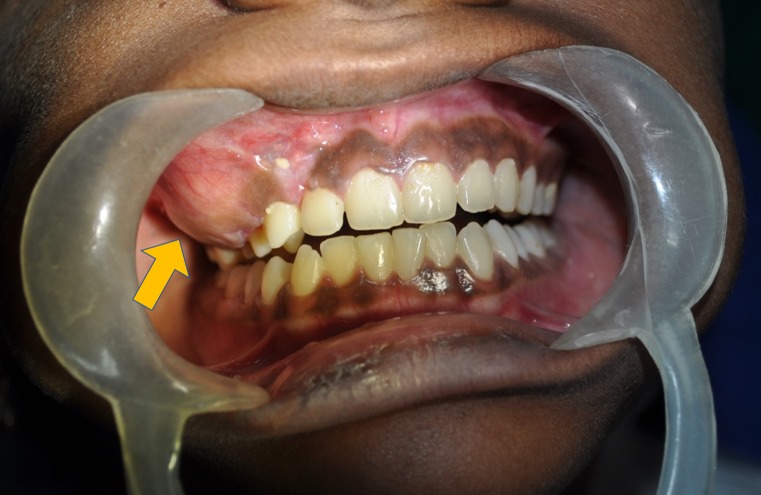
Intraoral view showing the swelling obliterating the upper right buccal vestibule from maxillary canine to the second molar (denoted by the yellow arrow).

The extraoral examination revealed diffuse swelling on the right side of the face, extending from right alar region to the infraorbital margin superiorly, and extending laterally to the right cheek region (Figure 1).The skin over the swelling was smooth. Palpation confirmed these findings. The swollen tissue had a normal temperature, was hard in consistency, nontender, and the overlying skin was pinchable. There was no paraesthesia or lymphadenopathy. The intraoral examination revealed an expansion of the right buccal cortex, which was hard on palpation (Figure 2). The swelling measured approximately 3 cm x 2.5 cm in the right maxillary region, obliterating the buccal vestibule. The overlying mucosa was normal without secondary changes. There was no evidence of tooth mobility or malocclusion, abscess formation, draining fistula or sinuses, or dehiscence.

**Figure 3:**
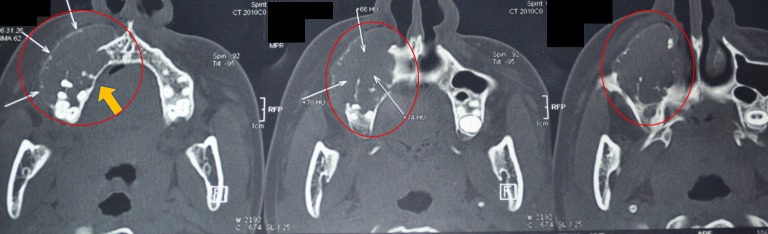
Computed tomography axial section shows expansion of the buccal cortex showing anteroposterior and labio-palatal extension (areas circled in red, denoted by the yellow arrow).

**Figure 4:**
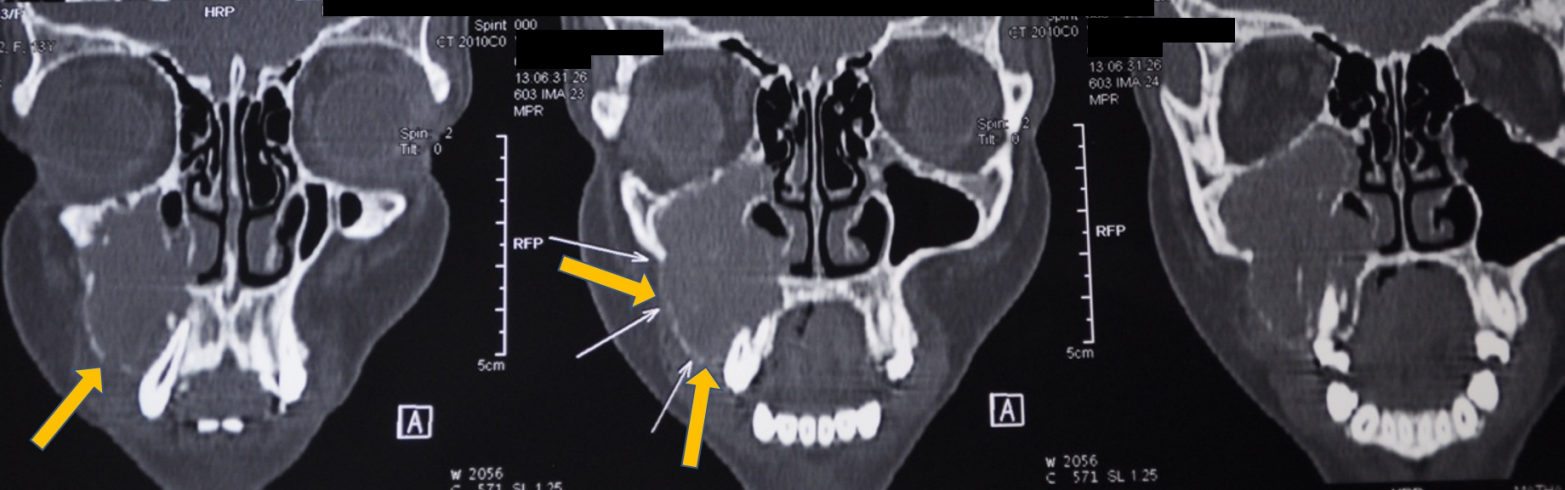
Computed tomography coronal section shows obliteration of the right maxillary sinus and superoinferior extension of the lesion (denoted by the yellow arrows).

**Figure 5:**
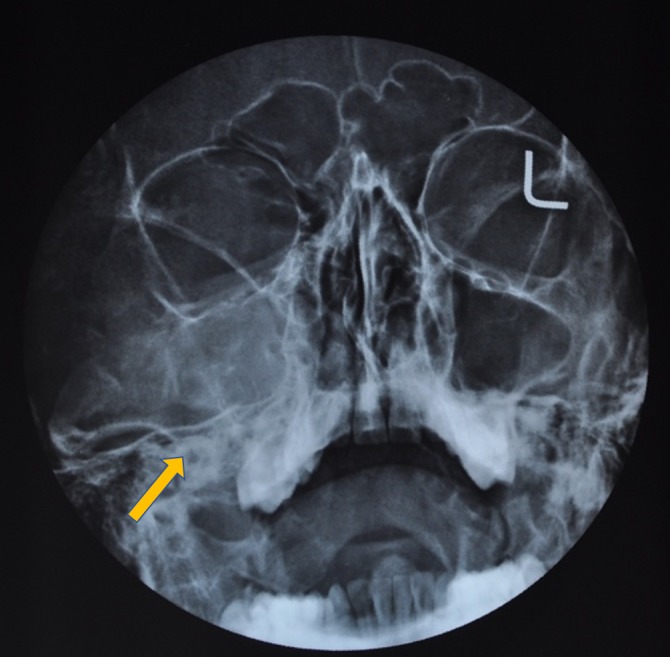
Plain paranasal sinus view showing mixed radiopaque/radiolucent lesion on the whole right maxilla and the maxillary sinus (marked by the yellow arrow).

The computed tomography scan of the right maxilla showed medial and lateral cortical expansion, with the lesion extending to the floor of the orbit (Figure 3, Figure 4). The paranasal sinus view had opacification of the right maxillary sinus (Figure 5). We also saw areas of central calcification with obliteration of the right maxillary sinus.

**Figure 6:**
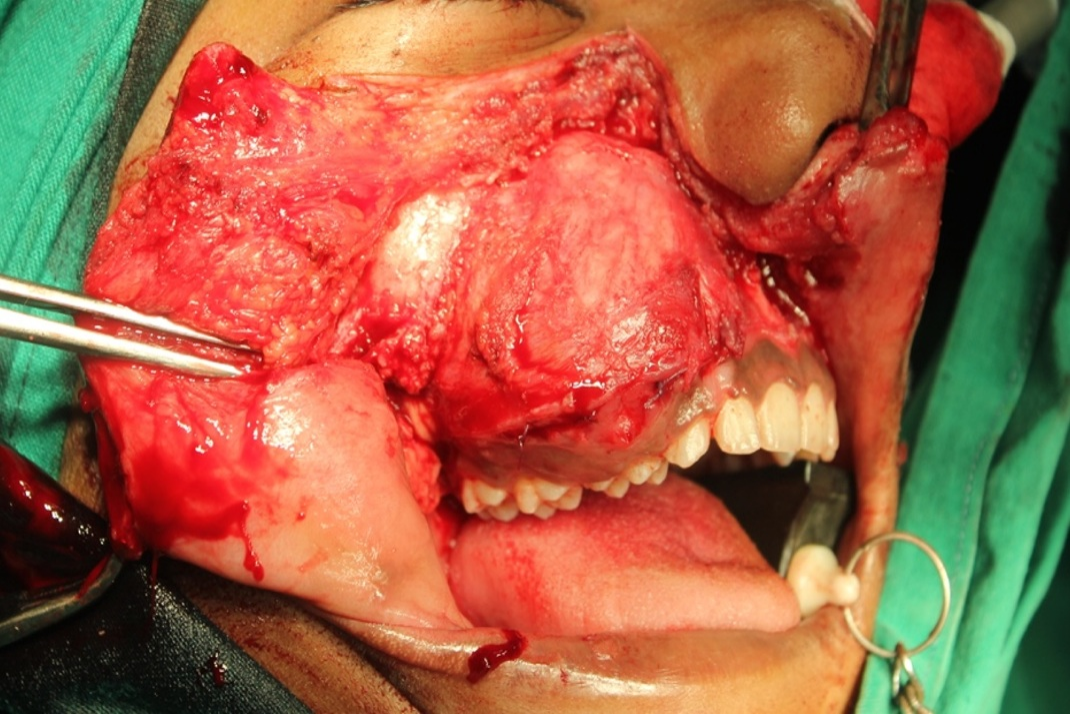
Intraoperative surgical access and exposure of the lesion using the Weber-Fergusson incision.

**Figure 7:**
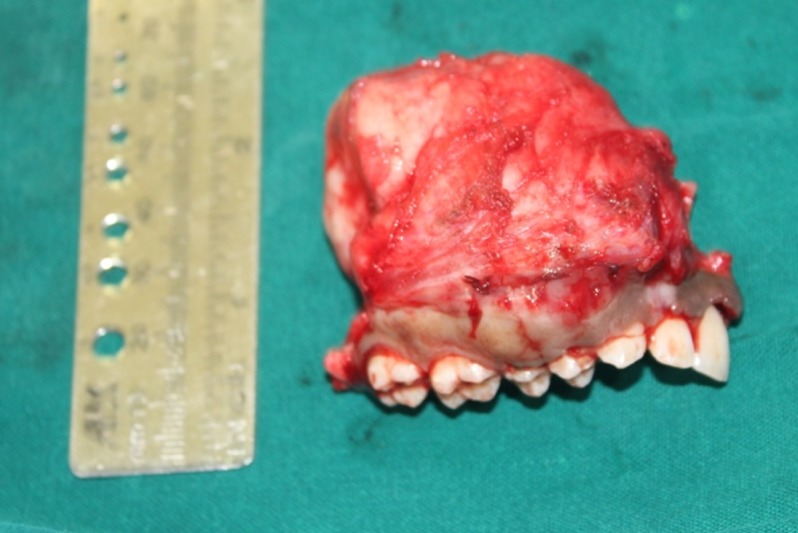
Excised specimen of maxilla measuring 6 cm x 5 cm x 5 cm.

We performed an incisional biopsy that confirmed the diagnosis of JOF. We then planned a maxillary resection with skin grafting. The patient underwent general anesthesia, and we placed a Weber-Fergusson incision on the right side to approach the maxilla (Figure 6). We resected the maxilla, sparing the floor of the orbit (Figure 7). A split-thickness skin graft was harvested from the right thigh region and secured on the denuded bone surface with sutures. The bolus dressing was placed using a surgical obturator. The excised specimen measured approximately 6 cm x 5 cm x 5 cm in size. The excisional biopsy report confirmed the incisional biopsy report.

**Figure 8:**
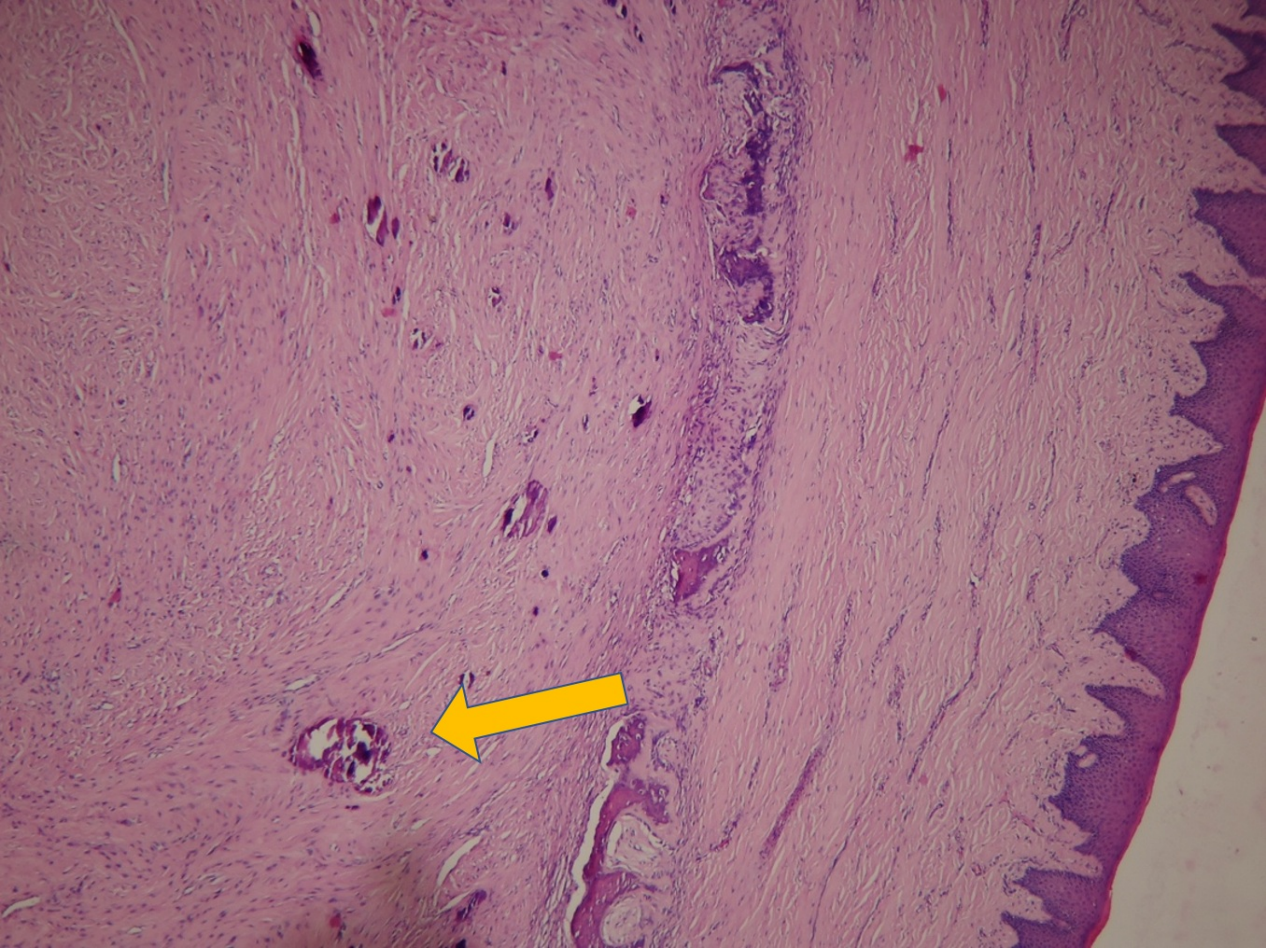
Hematoxylin and eosin stain showing histopathological juvenile ossifying fibroma (yellow arrow) under 10X magnification.

**Figure 9:**
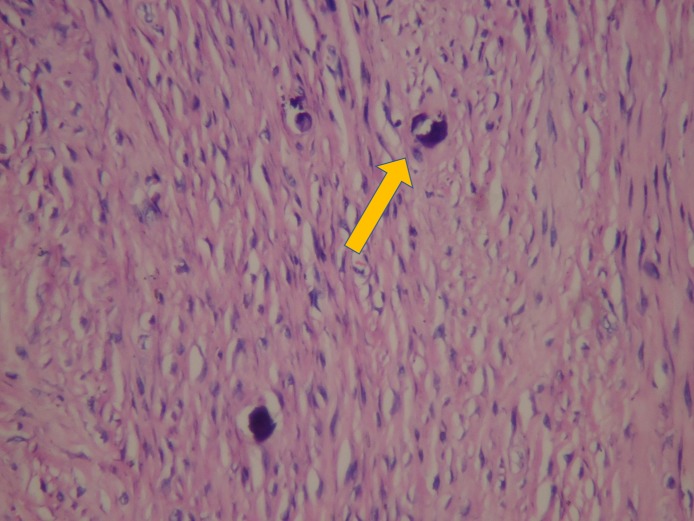
Hematoxylin and eosin stain showing histopathological juvenile ossifying fibroma (yellow arrow) under 40X magnification.

Histopathology with hematoxylin and eosin staining showed irregular strands of highly cellular osteoids encasing plump and irregular osteocytes as well as multinucleated osteoclasts (Figure 8, Figure 9). These findings confirmed the patient’s condition as TrJOF. 

## Discussion

The etiology of JOF is unknown. Suspected origins include traumatic, developmental, and odontogenic causes, and the periodontal ligament may also have a role in its etiology. Tumor suppressor gene HPRT2 has also been reported as a potential factor in JOF [2]. JOF affects young children under the age of 15, and its growth is aggressive and asymptomatic when it occurs in the maxilla. JOF occurs most frequently in the paranasal sinuses (approximately 90% of cases), whereas mandibular lesions account for approximately 10% of facial JOF cases [1]. There is no significant gender predilection, and most cases of JOF are asymptomatic, as seen in our case. The first clinical manifestation is a gradual, painless swelling of the affected bone (usually the maxilla). TrJOF seems to show more aggressive growth than PsJOF. In our case, the lesion had expanded to a large size without destroying adjacent vital structures, and without perforating the walls of the maxilla.

If the orbital bones or paranasal sinuses are involved, the patient may develop exophthalmos, bulbar displacement, nasal obstruction, and epistaxis [3]. Our patient’s blurred vision in the right eye could not be correlated with the lesion because there was no involvement of the right orbital floor or the optic canal.

Radiographically, lesions appear highly variable depending upon the stage of development and amount of calcification. JOF is characterized by three stages in radiographs. Stage I appears as a well-defined radiolucency with no evidence of internal calcification. Stage II (i.e., the mixed stage) is characterized by flecks of radiopacities in the radiolucent area. Stage III (i.e., the mature stage) is a completely radiopaque mass [2]. Erosion and invasion of the surrounding bone may also be noted in more aggressive cases. It usually takes at least six years for the lesion to pass from the radiolucent stage to the radiopaque stage. Therefore, radiolucent and mixed density lesions are usually reported. However, rapid maturation and complete radiopacity may occur in less than one year in very rare cases [4]. In our case, the lesion was in Stage II.

Microscopically, JOF is characterized by the presence of trabeculae of immature woven bone, cementum-like tissue, or both in a fibrous stroma. PsJOF is characterized by a proliferation of benign spindle‑shaped fibroblastic cells with embedded mineralized structures, which may present with round to ovoid collections of bone with an osteoid rim. Multinucleated osteoclast‑like giant cells may be seen with occasional normal mitotic figures, but atypia is not a usual feature. Our patient had TrJOF, which characterized by a fibroblastic spindle cell stroma containing osteoid matrix, surrounded by osteoblasts and anastomosing trabeculae of immature woven bone, often intermixed with scattered clusters of multinucleated giant cells; mitoses may be present, but cystic degeneration is rare [5].

JOF must be differentiated from malignant bone tumors other fibro-osseous lesions. The most prominent condition in the differential diagnosis is cemento-ossifying fibroma and fibrous dysplasia. The rapidity of growth, monostotic nature, and the well‑delineated radiographic margins differentiate JOF from fibrous dysplasia. Microscopically, cemento-ossifying fibroma contains cementicles [2], and while it is of odontogenic origin, it predominantly occurs in the second and third decades of life.

The recurrence rates after resection range from 30% to 56%. This may be due to the infiltrative nature of the tumor borders after an incomplete excision of the tumor, rather than any intrinsic biological properties of the tumor [6]. Despite the aggressive nature and high rate of recurrence, malignant transformation has not been reported. Aneurysmal bone cysts and hemorrhage are the secondary changes commonly associated with JOF. A complete excision of the tumor is the treatment of choice, as was done in our case. Though it is benign, JOF can be locally invasive, causing significant morbidity and mortality due to intracranial extension. JOF is radio-resistant, which means radiotherapy should be excluded as a treatment option [7].

## Conclusions

JOF is a benign neoplasm of the bone that has a potential for excessive growth, bone destruction, and recurrence. The treatment of choice is surgical excision, especially when JOF is located in the maxilla, and requires radical surgery with healthy margins. Early detection, complete surgical excision of these lesions, and long-term follow-up evaluations are important in the clinical management of JOF cases, due to its aggressive nature and high recurrence rate. The prognosis of JOF is good unless it recurs locally.
